# A Clinical Case

**Published:** 1884-07

**Authors:** Wm. Jefferson Guernsey

**Affiliations:** Philadelphia


### A CLINICAL CASE.

WM. JEFFERSON GUERNSEY, M. D., PHILADELPHIA.

On the 17th of February last I attended Mrs. W., aet. forty years, during her first confinement, and delivered her of an apparently healthy male child. On the morning of the 19th I learned that the child had had no evacuation of either bladder or rectum, and that during the night it had vomited what, at the time of my visit, looked like meconium, and that at both five and eight and a half o'clock A. M., had passed an enormous quantity of clotted blood. I examined it carefully and could not detect the least trace of meconium, nor was it at all viscid, and I am sure that it was nothing but blood, part of which was clotted and almost black. No urine had been voided, and there was already an emaciated look about the child. Lippe's Repertory, p. 126, gives:

"Blood, discharge of, from anus"—*Merc. cor.* (and many other remedies.)

Blood black—Ant. c., Merc. cor.

Blood clotted—Merc. cor., Stram.

Merc. cor. also has suppression of urine and vomiting of "blood like coffee grounds, and coagulated blood." (Not an exact description of the vomit but a *similar* one.) ℞ 4 powders Merc. cor., 45m. (F.) on every two hours.

At three and a half P. M. had slight discharge of blood, another still smaller in quantity, at about midnight, and a few drops of blood at 1.30 P. M. the next day (20th). The little boy had urinated during the night but the nurse could not tell exactly when. As each hæmorrhage succeeding the administration of the medicine had been lighter, no more medicine was given. The child has remained perfectly well to the present time, is growing finely, and its bowels and kidneys are normal in their action. Another item of interest, however, remains to be told: On the 6th of March the nurse found the little boy's breast full of a substance resembling milk and containing so much of the fluid as to be quite hard and at the same time "weeping" considerably.

I gave three powders of Phytol. 5c (T.) and had the satisfaction of finding each mammilla soft, and much smaller and without discharge in less than half a day. No recurrence of either phenomenon to date.

